# Carotid MR angiography using pulsed continuous arterial spin labeling

**DOI:** 10.1186/1532-429X-13-S1-P371

**Published:** 2011-02-02

**Authors:** Koktzoglou Ioannis, Robert R Edelman

**Affiliations:** 1 NorthShore University HealthSystem, Evanston, IL, USA

## Introduction

Carotid artery disease is a major cause of cerebral ischemia and infarction. It is routinely assessed using contrast-enhanced magnetic resonance angiography (MRA). Several methods for nonenhanced MRA exist, which may be particularly advantageous in patients with impaired renal function due to the risk of nephrogenic systemic fibrosis. Pulsed arterial spin-labeled (ASL) MRA has been investigated for carotid MRA, however, the signal to noise ratio (SNR) with the method is problematic when short repetition times are used to minimize scan time or when long labeling times are used to maximize visible vessel length. In contrast to pulsed ASL (PASL), we hypothesized that pulsed continuous ASL (PCASL) would provide improved SNR at short repetition times and/or long labeling durations.

## Purpose

To investigate the performance of pulsed continuous ASL (PCASL) for carotid MRA, with comparison to conventional pulsed ASL (PASL).

## Methods

This study was approved by our institutional review board. PCASL and PASL carotid MRA were compared in 5 volunteers (10 carotid arteries) using a 1.5 T scanner (Avanto, Siemens). Imaging was performed using a coronal 3D balanced steady-state free precession slab centered at the carotid bifurcations. Common imaging parameters were: field of view of 256x320 mm, spatial resolution of 1x1mm, 64 1-mm-thick slice after interpolation, repetition times of 1.2-2.0s, acquisition times of 98s-164s, labeling times of 0.9s-1.7s, parallel imaging (GRAPPA) acceleration of 3. Arterial signal measurements were obtained from the PCASL and PASL image sets, and the ratio PCASL to PASL signal was computed.

## Results

**Figure 1:**
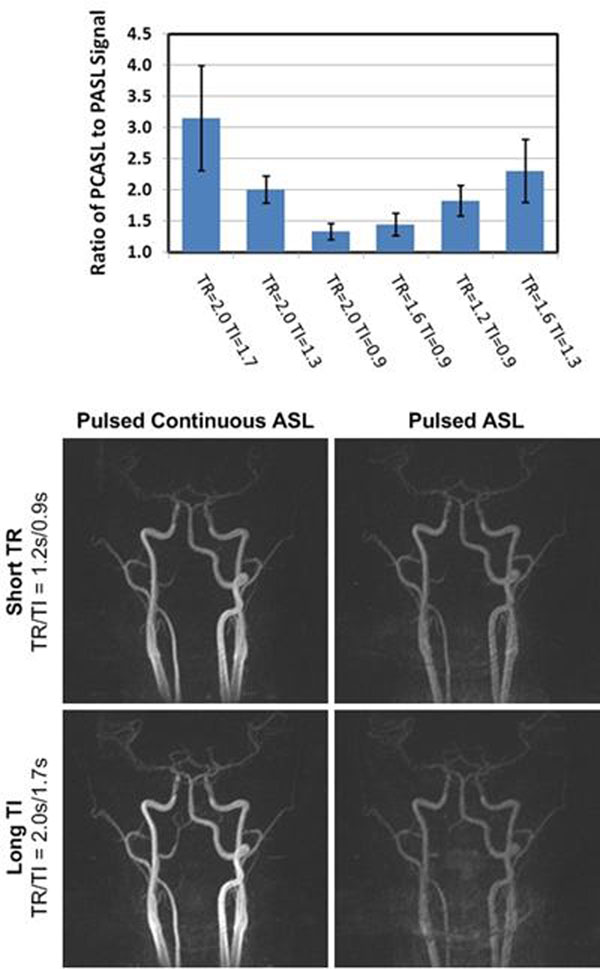
*Top panel*, Bar plot showing the ratio of PCASL to PASL signal in the carotid arteries. *Bottom panel*, montage of PCASL and PASL images acquired using short repetition and long labeling times. Images are shown using the same window and level settings. TR = repetition time; TI = labeling time/duration.

PCASL provided significantly larger arterial signal measurements than PASL for all repetition and labeling times investigated (Figure 1, top panel) (P < 0.05). The PCASL method improved the clarity with which the carotid arteries were depicted, especially when short repetition times (TR = 1.2s) and long labeling durations (TI = 1.7s) were used (Figure 1, bottom panel).

## Conclusion

Compared with pulsed ASL, pulsed continuous ASL is advantageous for carotid MRA because it maximizes vascular signal and minimizes the adverse effects of short repetition or long labeling times.

